# Health sciences students’ perception of the communicative impacts of face coverings during the COVID-19 pandemic at a South African University

**DOI:** 10.4102/sajcd.v69i2.890

**Published:** 2022-07-27

**Authors:** Nasim B. Khan, Nolwazi Mthembu, Aishwarya Narothan, Zamahlase Sibisi, Qiniso Vilane

**Affiliations:** 1 Discipline of Audiology, Faculty of Health Sciences, University of KwaZulu-Natal, Durban, South Africa

**Keywords:** communicative impacts, face coverings, COVID-19 pandemic, perceptions, challenges

## Abstract

**Background:**

The use of face masks and/or shields can pose a challenge during communication. They block facial expressions thus removing visual cues and affect sound transmission making it difficult to hear speech clearly. Given the widespread use of face coverings, it seems reasonable to clarify if communication in typical speakers and listeners has significantly differed. Health science students as future practitioners need to understand challenges that arise from using face coverings.

**Objective:**

This study aimed to determine health sciences students’ perception of the communicative impacts of face coverings.

**Method:**

The study employed a descriptive, self-administered online survey, obtaining information from 96 health science undergraduate students.

**Results:**

All participants changed their manner of speaking in that they spoke louder when wearing masks and focused more on eye contact when someone was wearing masks. These were statistically significant (*p* = 0.450 and *p* = 0.035 respectively). Fifty-three percent reported using more listening effort and feeling anxious when communicating. Approximately 33% indicated that it was challenging to read emotions, such as sad or unhappy, when someone wore a mask. Most, 61%, were positive or very positive about wearing masks. The level of difficulty differed depending on the listening environment. It was harder to understand the doctor, nurse, or other healthcare workers when they wore face coverings than when listening to their friends and family, which had little effect, this being statistically significant (*p* = 0.025).

**Conclusion:**

Challenges envisaged in practice included frequent communication breakdowns, inability to connect and build trust between patient and practitioner, and communicating in noisy environments. Coping strategies, future clinical and research implications were proposed, and limitations acknowledged.

## Introduction

The COVID-19 pandemic has rapidly transformed the delivery of healthcare services globally. Everyone has to wear a mask or face covering (Chodosh, Weinstein & Blustein, 2020). The use of face masks or personal protection equipment (PPEs) is encouraged and made mandatory in some countries for all their citizens and healthcare professionals because of the risk of rapid transmission of COVID-19 in healthcare settings (Lima, Simões, Taguchi, & Araújo, 2020; WHO, 2021). Furthermore, face coverings have become essential in public areas if social distancing is impossible (Saunders, Jackson, & Visram, 2020); however, in South Africa both interventions are recommended. Different types of face masks, including but not limited to N-95 masks and surgical masks, and various face shields, are utilised by healthcare practitioners to prevent and control the spread of the virus. This has proven to be critical in curtailing the spread of the virus (Corey, Jones, & Singer, 2020; Saunders et al., 2020). Wearing face masks, social distancing, proper hygiene, proper disposal of waste products and contact tracing measures are essential components of infection control (Magee et al., 2020; Nguyen et al., 2021; Saunders et al., 2020; Sharma et al., 2020). The control measures and protective benefits to curb the virus are well understood and well established; however, less is known about the potential barriers of masks and shields on communication.

Communication is the transmission of messages from one person to another (Vermeir et al., 2015). This includes verbal and nonverbal interpersonal communication such as eye contact, facial expressions and body language, illustrating empathy, sincerity and emotions (Murphy, Lewis & Gormley, 2021; Saunders et al., 2020). Face-to-face conversations are beneficial for an entire discussion and the proper meaning behind words using body language and facial expressions (Kratzke, Rosenbaum, Cox, Ollila, & Kapadia, 2021). Effective communication is vital in healthcare settings, both between a practitioner and a patient and among practitioners themselves. Communication is at the forefront of the patient–practitioner relationship, which is crucial for forming trusting relationships, explaining complex concepts and involving patients in joint decision-making, while considering their expectations and concerns (Kratzke et al., 2021). Expressing emotions and exchanging nonverbal communication are essential foundations for practitioner–patient clinical encounters (Houchens & Tipirneni, 2020). A trusting practitioner–patient relationship is associated with increased patient satisfaction, patient adherence and a higher quality of life (Kratzke et al., 2021). Using face masks and shields also creates barriers for empathetic communication, which is essential between healthcare workers and patients. Empathetic communication builds trust, which is crucial in healthcare settings and for achieving healthcare goals. Essentially, practitioners are seen as less empathetic when a face covering is used (Ten Hulzen & Fabry, 2020). In a study by Kratzke et al. (2021), only 22% of patients reported complete trust in their surgeon’s decisions when the surgeon donned a covered mask versus a clear mask. Extensive use of face masks, especially during the pandemic, also poses a challenge for healthcare practitioners working with paediatric caseloads. A study by Shack, Arkush, Reingold and Weiser (2020) found that half of the children aged 4–10 years preferred doctors using only face shields compared with masks, with some children even citing that they feared doctors wearing masks.

Nonverbal communication helps to build trust in therapeutic relationships that lead to improved clinical outcomes (Houchens & Tipirneni, 2020). In addition to this, all medical information should be reliable, unambiguous, accurate and well understood by professionals and patients. Viable communication between medical healthcare workers and the patient or their family is fundamental to guarantee that medical care is conveyed adequately and delivered effectively and efficiently (Ten Hulzen & Fabry, 2020).

Poor communication in healthcare settings can have various negative impacts, such as compromising the safety of patients, patient dissatisfaction, discontinuity of care and the inability to use valuable resources efficiently (Vermeir et al., 2015). Conversely, patients who hear and understand their healthcare provider will likely comply with treatment and follow recommendations appropriately. Poor communication may lead to miscommunication between health practitioners and patients. In most cases, the underlying cause for miscommunication is an age-related problem as older people suffer from hearing loss; however, it has received little attention (Blustein et al., 2020). Health care is also delivered in extremely noisy and distracting settings for patients with alarms beeping, multiple activities simultaneously and ongoing competing conversations between patients and providers. Some people speak softer than others and can often not be heard because of ambient environmental noise around them. Increased effort is thus needed in listening strategies (Ten Hulzen & Fabry, 2020). Communication can be challenging in loud and very noisy environments (Magee et al., 2020). Multiple distractions, often with background noise with a lower signal-to-noise ratio (SNR), significantly hinder communication (Magee et al., 2020). Hospitals are sites where communication is critical and vital. The challenging listening situations described above are more difficult for people with hearing loss to understand speech. This creates a barrier to care that leads to a communication disadvantage compared with normal hearers (Martin, 2020).

Speech intelligibility is further compromised when face coverings block complementary visual cues (Magee et al., 2020), making it difficult to perceive emotions such as happiness, anger, or disgust and impairing facial recognition and identification (Naylor, Burke, & Holman, 2020). The inability or reduced ability to view visual cues, facial expressions and failure to lip-read the other person, necessary to supplement verbal information, make it very difficult to hear and comprehend speech and deliver the intended message (Kratzke et al., 2021; Mheidly, Fares, Zalzale, & Fares, 2020; Saunders et al., 2020). This is more pronounced in individuals with a hearing loss who rely on visual cues to understand others effectively (Kratzke et al., 2021; Saunders et al., 2020), an approach lost once the face shield or mask is used (Houchens & Tipirneni, 2020).

Research evidence has shown that face masks and shields can interrupt sound transmission, thereby affecting speech signal quality and clarity (Magee et al., 2020; Nguyen et al., 2021; Saunders et al., 2020; Sharma et al., 2020). Atcherson et al. (2017) reported that higher frequencies are dampened when speaking through a face mask. Higher frequencies aid in differentiating similar sounds; however, masks muffle speech sounds, which hinders speech perception, particularly in environments that are very noisy or when someone has a hearing loss (Hampton et al., 2020). The acoustic effect of someone speaking while wearing a face mask and the resultant voice distortion because of attenuation is comparable with the listener experiencing a slight high-frequency hearing loss (Chodosh et al., 2020; Corey et al., 2020; Gaeta, 2020).

The type of masks worn can also contribute to the lack of speech intelligibility and degradation of speech signal (Nguyen et al., 2021). The N95 respirators impact speech understanding by listeners, with word intelligibility dropping between 1% and 17% (Nguyen et al., 2021). The study carried out by Nguyen et al. (2021) showed that a mask’s filtering ability and its features also determine the voice level, although the degree to which a speaker wearing a mask adjusts his or her phonation style may differ.

A few studies have investigated the effect that surgical masks have on speech perception among healthcare workers in their work environment. Bandaru et al. (2020) assessed the impact of using an N95 mask and face shield on speech perception among healthcare workers with normal hearing in India. The results revealed an increase in speech reception threshold (mean of 12.4 dB) and a decrease in speech discrimination score (mean of 7%), with this being statistically significant, demonstrating that these coverings impair speech perception. However, the study was conducted in a sound-treated room without any external noise. In hospitals, there is a much background noise; hence, further research needs to be undertaken with background noise to conclude how much worse the situation can be.

Goldin, Weinstein and Shiman (2020) measured the listener’s comprehension at the acoustic level presented by the different surgical masks worn by the healthcare workers. Acoustic degradations were reported whereby medical masks act as low pass filters. For simple surgical masks, high frequencies in the range of 2000–7000 Hz are attenuated by about 3–4 dB, and for N95 masks up to 9–12 dB – the latter results support the study by Bandaru et al. (2020). Furthermore, developments in another study showed that speech quality degradation in an environment with noise and not being able to lip-read because the mask has covered the lower part of the face causes greater difficulty in understanding speech (Nguyen et al., 2021).

Similar to the Goldin et al.’s (2020) study, Saunders et al. (2020) found that masks act as low pass filters, attenuating sounds above 2 kHz. These masks can reduce sound intensity up to 20 dB, with the least attenuating masks being surgical masks, decreasing sound by 2–4 dB. Martin (2020) also reported reduced sound levels at high frequencies. Thus, surgical masks may not have that great impact on speech intelligibility. The listeners understand speech irrespective of background noise or whether the listener had a hearing loss (Saunders et al. 2020).

A study was conducted by Toscano and Toscano (2021) to determine mask effectiveness among different types of masks on speech recognition in various levels of background noise. Two cloth masks, an N95 respirator mask and a surgical mask, were used. The surgical masks produced the slightest effect of speech recognition reduction, similar to not wearing masks. The cloth masks yielded the worst effect where poor speech recognition results were obtained. It was noted that face masks have a small impact on speech recognition at low noise levels. At high noise levels, the effects of the different types of masks were visibly noticed as the cloth masks, and an N95 respirator revealed the poorest speech recognition thresholds (Toscano & Toscano, 2021).

A study by Giovanelli, Valzolgher, Gessa, Todeschini and Pavani (2021) aimed to clarify the impact of face masks on speech understanding starting from one of the most common communication modalities imposed by social distancing, namely, video calls. Speech comprehension was tested in typical hearing participants while conducting video calls with talkers in three different conditions: with a mask on, without a mask on and, their name only appearing on the screen. Lower performance was observed when only the name appeared on the screen or when the lips were concealed with a mask. This was accompanied by lower confidence scores and increased perceived effort, demonstrating that face masks impact speech comprehension, whether in person or via a video call (Giovanelli et al., 2021).

Speech audibility is also negatively affected by social distancing as sounds become softer as they travel away from the source (Ten Hulzen & Fabry, 2020). Because of speech audibility being affected, communication and interaction between people are also affected, and hence distance issues have been created, causing communication barriers (Ten Hulzen & Fabry, 2020). While speech perception accuracy worsens at longer distances, speaking with a mask exacerbates the situation (Nguyen et al., 2021). Thus, multiple factors affect communication, and the effect’s magnitude depends on the speaker, type of mask worn, the listener’s hearing ability, visual cues available and background noise (Nguyen et al., 2021; Spitzer, 2020). This can then range from negligible to considerable effects (Nguyen et al., 2021).

Healthcare practitioners thus need to understand the impacts of face coverings when they speak or listen to their patients who present with any condition, disorder or disability. Furthermore, healthcare practitioners must be aware of the challenges faced by persons with hearing loss to understand speech, exacerbated by face coverings. Individuals with hearing loss require various services as they may need to have an eye test, need to get medication from a pharmacist and so forth. Thus, this understanding among different professionals is essential, given that the dire consequences of hearing loss on clinician–patient communication, substantially magnified with the pandemic (Chodosh et al., 2020). Because of the circumstances caused by COVID-19, patients who may need to arrive with family members and interpreters would be restricted from the healthcare setting, which causes detrimental impacts on the quality of health care provided and communication. Communication can be misinterpreted easily when the healthcare professional and the patient use universal masking. If one or both of them have a hearing loss, there would be reliance on oral communication, leading to significant negative impacts (Ten Hulzen & Fabry, 2020). This can lead to practitioners experiencing communication stressors because interpersonal communication is negatively affected, with face coverings obscuring the mouth, limiting visual cues, and reducing understanding (Campagne 2021). A study by Heider et al. (2021) that aimed to determine the prevalence of voice disorders in healthcare workers of high-risk hospital care units found that nearly 33% of healthcare personnel reported voice disorders during the pandemic period; this being higher than the prevalence of voice disorders in general population at any other time which is around 7%. This was exacerbated by long work hours and increased use of masks during COVID-19. To obtain speech understanding with 90% accuracy, the signal above the noise source should be around 10 dB–15 dB (Way et al., 2013 cited in Heider et al., 2021). Thus, with an estimated background noise level of 65 dB sound pressure level (SPL), health personnel to be understood with 90% accuracy would have to speak at levels of 80 dB SPL (Way et al., 2013 cited in Heider et al. 2021). Thus, regardless of the purpose of use, the face mask is perceived to lead to vocal fatigue, discomfort and communication difficulties. This is particularly important to be considered in individuals who use face masks for professional and essential healthcare-related activities (Nguyen et al., 2021).

Communication is a crucial aspect of the interaction between people and occurs daily. Understanding communication while wearing a mask is essential. Communicating clearly and naturally is necessary for accurately understanding speech requiring less listening effort than if speech is in any way degraded (Nguyen et al., 2021). From a user’s perspective, face coverings can also mean increased vocal effort and reduced auditory feedback (Nguyen et al., 2021). Face masks and face shields cause a hindrance as the message that is sent between people is often miscommunicated (Chodosh et al., 2020). Given the current widespread use of face masks, it seems reasonable to clarify whether speech and hearing in healthy speakers and listeners have significantly differed with face coverings.

This study aimed to determine the perception of health sciences students on the communicative impacts of face coverings during the COVID-19 pandemic. To meet the aim of this study, the following objectives were devised: (1) to determine health science students’ perceptions of the communicative impact of face coverings during the COVID-19 pandemic as *speakers*, (2) to determine health science students’ perceptions of hearing and communicative impact of face coverings during the COVID-19 pandemic as *listeners,* and (3) to determine health science students’ perceptions of the communicative impact of face coverings during the COVID-19 pandemic in a *clinical setting.*

## Methods and materials

### Research design

A quantitative descriptive research approach was used. This type of design is based on the standard of gathering information regarding the situation at hand and describing the relationship between variables (Hulley, Cummings, Browner, Grady, & Newman, 2007). A descriptive survey describes events, phenomena and attitudes towards a problem. These studies typically involve one contact with the study population and are reasonably cheap and less time-consuming to undertake (Kumar, 2005; Lodico, Spaulding, & Voegtle, 2010).

### Study sample, sampling technique and sample size

This study was conducted with undergraduate students from the School of Health Sciences (SHS) at the University of KwaZulu-Natal, Westville campus. All students from first to final year registered in the SHS were invited to participate in the study through the university intranet notice system. The included disciplines were Audiology, Pharmacy, Speech-Language Pathology, Optometry, Physiotherapy, Occupational Therapy, Dental Therapy or Dentistry, and Sports Science. The statistician consulted from the College of Health Sciences utilised the GPower software version 3.1.9.7 for sample size calculation. It was estimated that a sample size of at least 96 participants was required to detect an effect size of 0.5 about 80% of the time. According to the statistician, the sample size is the minimum number needed based on the study type, questions and required responses.

All first- to final-year students, from the age of 18 years and above, irrespective of gender, or ethnicity, registered in the disciplines mentioned above were included in the study. While first-year students do not have any clinical blocks, they still need to understand the impacts of face coverings to apply this to their clinical settings. While it was envisaged that audiology and speech-language pathology students might be a bit more knowledgeable about communication, wearing masks is unusual for all students in this current pandemic, and they were included. Students below 18 years and who are not currently registered in the SHS were excluded from the study. A convenience sampling technique was utilised, allowing for a larger sample to be analysed as the required information is also acquired quickly. Participants were selected based on accessibility, availability, flexibility and feasibility (Leedy & Ormrod, 2015; Maxwell & Satake, 2006). The demographic information obtained from the study revealed that most of the them, 85.3% (*n* = 82), were female participants at the age of 21 years, 36.5% (*n* = 35). The majority, 24.0% (*n* = 23) participants, were from the discipline of Audiology, followed by Physiotherapy, and from the third year of study, 42.7% (*n* = 41). Most (75.0%, *n* = 72) of the students’ first language was isiZulu, with 81.3% (*n* = 78) indicating their ethnicity as African. Table 1 summarises the demographic information of the participants.

**TABLE 1a:** Summary of the demographic information of the participants.

Variables	Demographic information (*n* = 96)								
Age	18	19	20	21	22	23	24	25	> 25
%	3.1	8.3	16.7	36.5	17.7	3.1	7.3	2.1	5.2
*n*	3	8	16	35	17	3	7	2	5

**TABLE 1b:** Summary of the demographic information of the participants.

Variables	Demographic information (*n* = 96)							
Discipline of study	Sport science	Dental therapy	Occupational therapy	Pharmacy	Physiotherapy	Optometry	Audiology	Speech-language therapy
%	2	15.6	3.1	11.5	22.9	9.4	24	11.5
*n*	2	15	3	11	22	9	23	11

**TABLE 1c:** Summary of the demographic information of the participants.

Variables	Demographic information (*n* = 96)				
Year of study	1st	2nd	3rd	4th	Other
%	6.2	18.8	42.7	31.3	1
*n*	6	18	41	30	1

**TABLE 1d:** Summary of the demographic information of the participants.

Variables	Demographic information (*n* = 96)				
Ethnicity	African	White	Coloured	Indian	-
%	81.3	2.1	1	15.6	-
*n*	78	2	1	15	-

**TABLE 1e:** Summary of the demographic information of the participants.

Variables	Demographic information (*n* = 96)				
Home language	English	Isizulu	Afrikaans	Other	-
%	20.8	75.0	1.0	3.2	-
*n*	20	72	1	3	-

**TABLE 1f:** Summary of the demographic information of the participants.

Variables	Demographic information (*n* = 96)				
Gender	Male	Female	-	-	-
%	14.7	85.3	-	-	-
*n*	14	82	-	-	-

### Data collection and analysis

In order to facilitate data collection, a survey questionnaire was used, which was adapted from Saunders et al. (2020) and Nguyen et al., (2021). The questionnaire included 25 questions with both open-ended and closed-ended questions, together with Likert scale options. Section A comprised of demographic information, Section B included perceptions of the communicative impact of face coverings during the COVID-19 pandemic as *speakers*. Section C included perceptions of the communicative implications of face coverings during the COVID-19 pandemic as *listeners*. Finally, section D included perceptions of the communicative impact of face coverings during the COVID-19 pandemic *in a clinical setting.*

Using an online questionnaire minimised the printing costs and saved much time distributing and collecting questionnaires. Online survey questionnaires have been deemed to be the upcoming preferred research method as they will allow researchers to gather large quantities of data effectively without exceeding monetary constraints (Lefever & Dal, 2007). Online surveys also lower the probability of data and interviewer errors by eliminating the potential of human error during the manual entry. Moreover, online surveys are self-administered. The participants can often commence and pause at their convenience, and it can be completed at any desired location (Lumsden, 2007). However, limitations of online survey questionnaires include low response rates and incomplete responses.

A notice was sent via the university web notification system to all SHS students requesting their participation in the study with a link to Google forms. Participants were given a link to Google forms, asking for their consent to participate in the study. They received additional information on the research and what was required from them. The participants were asked to complete the questionnaire in three weeks. After three weeks, an additional two weeks were given to students to complete the questionnaire for improving the response rate. Regular reminders were posted on the university notice system to remind students to participate. The study was kept open for a total of six weeks. By word of mouth, the respective class representatives were also asked to help in sharing the online link with their classmates and encouraging them to participate in the study.

While the recruitment letter indicated that the study was only targeting undergraduate students, three post-graduate students answered the questionnaire; however, their results were not included in the study analysis. A pilot study was conducted with five participants, each from a different discipline, before the commencement of the primary research. The intention was to get one participant from each of the eight fields; however, only five participants from five different disciplines responded to the pilot study. The student researchers accessed the pilot participants through word of mouth and personal contact with fellow students to participate voluntarily. The documents were made available to these pilot participants on Google Forms, including an information document, consent form, questionnaire, and a pilot feedback form for the questionnaire. In addition to the comments on the questionnaire’s content, the participants were asked to indicate if the questionnaire was of appropriate length, suitable language, and if the time taken to complete the questionnaire was adequate.

All five pilot participants suggested that the language was reasonably straightforward to understand. Most felt the time was right at 15 minutes to complete the questionnaire. Three participants thought it was okay, that is, neither too long nor too short. Four participants felt the questions were clear, and there was no ambiguous question. Two participants felt that the questionnaire was a bit too long and repetitive and made it difficult to follow, especially Q17, and the last six questions seemed to be repeated. These were adjusted by combining four questions into two questions and rephrasing some of the options to make it easier to follow. The information and feedback obtained from the pilot study enabled the researchers to make necessary adjustments as stated above. The results of the pilot study were not included in the main study.

The findings of this study were analysed using both descriptive and inferential statistics. Data were coded, entered on Excel, and then exported to SPSS (V27). The descriptive statistics were displayed in the form of frequencies and percentages. The results were represented graphically in charts, graphs and tables. The study tested independent associations between two categorical variables. The chi-square test or Fisher’s exact test depending on the frequency distribution was used to evaluate these associations. Examples of associations include gender, year and level of study versus the perceptions as speakers, listeners and envisaged challenges in clinical settings. The confidence level was set at 95%, with a significance level of 0.05. The above data analysis was carried out in consultation with a statistician from the College of Health Sciences.

### Reliability and validity

Cronbach’s alpha was utilised to ensure reliability and to test the study’s internal consistency. The purpose of Cronbach’s alpha is to estimate reliability from correlations obtained from the data collected within the questionnaire (Andrew, Pedersen, & McEvoy, 2011). The overall Chronbach alpha score received was 0.836 for the questionnaire based on 37 individual items within the questionnaire. According to Biddle (2006), the reliability of normative data, 0.8 to 0.89, demonstrates good reliability and good internal consistency. Efforts to improve the reliability and internal validity of the study included conducting an in-depth review of the literature in order to ensure that the research methodology was aligned with other studies. A pilot study was undertaken to ensure that the questionnaire and online system were appropriate for this study. External validity refers to how the results obtained from the study apply to contexts beyond the proposed study population (Leedy & Ormrod, 2015). Due to the type of sampling techniques and sampling effects such as inadvertent selection bias, it was envisaged that results might not be generalised to other contexts.

### Ethical considerations

Prior to data collection, ethical clearance was obtained from the Humanities and Social Sciences Research Ethics Committee, University of KwaZulu-Natal (reference number: HSSREC/00002749/2021). Gatekeeper permission was granted by the Registrar of the University. The researchers completed a web-based ethics training course from the Training and Resources in Research Ethics Evaluation (TRREE). Participants were made aware that their participation is voluntary, and they can withdraw from the research study at any point (Bhattacherjee, 2012). Anonymity indicates that the researchers who have completed the final report cannot identify specific responses to a particular participant. No personal information was collected as the researcher had no way of knowing which participant responded, and the email address was not linked. Confidentiality involves the researcher’s promise that they will refrain from divulging a participant’s identity in the report, even if they can identify the response (Bhattacherjee, 2012). The above attempts to ensure participant anonymity and confidentiality is consistent with the *Protection of Personal Information Act (POPIA) (4 of 2013)* as enshrined in Section 14 of the South African Constitution. The online data obtained will be password protected and kept safely in a locked cabinet in the University’s Audiology Department for five years and will only be available to the researchers and their supervisor until the research has been completed. After that any hard copies will be destroyed by shredding and the online data by deleting.

## Results

The results are presented according to the three objectives of the study. All the descriptive statistics are given; however, only some significant associations are shown for the inferential statistics.

### Objective 1

To determine health science students’ perceptions of the communicative impact of face coverings during the COVID-19 pandemic as speakers.

The participants were given two general questions about their self-reported hearing ability and attitudes towards wearing masks and shields. The majority, 78% (*n* = 75), reported that their hearing was good (42%) or very good (36%). Of the remaining participants, 17% stated that their hearing was average and 5% poor or very poor. Most 61% (*n* = 59) were positive about using masks, 31% (*n* = 30) were neutral, and a few participants (*n* = 7) were negative or very negative about wearing masks. Most participants who were generally negative or neutral about wearing masks stated that they pull their face mask down when they feel that the other person cannot hear or understand them, and this was statistically significant (*p* < 0.001) (Pearson Chisq. test).

**FIGURE 1:**
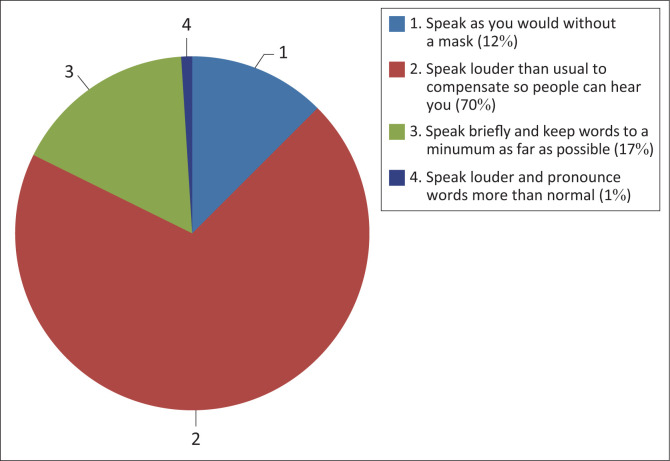
Speakers adjust content, manner and length of utterances when using face coverings (*n* = 96).

Participants had to provide information on how face masks or shields change the manner in which they speak, how the content of their conversation is affected, how the length of their utterances is affected and information on their interpersonal communication. With regard to the length of utterances that were affected by face masks or shields, the majority of participants, 70% (*n* = 67), speak louder than usual to compensate for the face masks or shields so that people hear them; 17% (*n* = 16) of participants speak briefly and keep words to a minimum as far as possible as depicted in Figure 1. Regarding the language spoken, all participants stated that they changed their manner of speaking in that they speak louder, with increased pitch, when wearing masks, which was statistically significant (*p* = 0.450; Fisher’s exact test).

With regard to interpersonal communication, about 49.0% (*n* = 47) were sometimes even more expressive with their voice when speaking using face masks that they would without, and 46.9% (*n* = 45) sometimes used bigger hand gestures and movements when speaking using face masks (see Table 2). The majority of them, 71.9% (*n* = 69), focused more on eye contact when speaking using face masks, to check whether people were listening to them. There was a statistically significant association between gender and use of hand movements and eye contact (*p* = 0.012; Pearson Chisq. test) (*p* = 0.035; Fisher’s exact test), respectively, with more women, indicating that they use bigger hand movements and more eye contact when communicating. However, results must be viewed with caution given the male-to-female ratio of 1:5.6 in this study.

**TABLE 2:** Summary of interpersonal communication.

Interpersonal communication	No		Sometimes		Yes	
	Number	%	Number	%	Number	%
Are you more expressive with your voice when speaking using face masks?	29	30.2	47	49.0	20	20.8
Do you use bigger hand gestures and movements when speaking using face masks?	20	20.8	45	46.9	31	32.3
Do you focus more on eye contact when speaking using face masks to check if people are listening to you?	11	11.5	16	16.7	69	71.9
Do you feel more self-conscious when speaking using face masks?	39	40.6	28	29.2	29	30.2
Do you pull your face mask down when you think that the other person cannot hear or understand you?	24	25.0	33	34.4	39	40.6
Do you overcompensate and exaggerate speech to make it more intelligible to get someone to hear or understand you?	15	15.6	49	51.0	32	33.3
Do face masks and shields make it difficult to see facial expressions?	4	4.2	19	19.8	73	76.0
Do you believe that it is difficult for you to be heard clearly by others when you are wearing a mask?	5	5.2	33	34.3	58	60.4

When asked how they felt about wearing masks, 40.6% (*n* = 39) did not feel more self-conscious when speaking using face masks. However, 30.2% (*n* = 29) of participants felt more self-conscious when speaking with face masks. Most of them, 40.6% (*n* = 39), pull their face mask down when they think that the other person cannot hear or understand them, and 51.0% (*n* = 49) sometimes overcompensate and exaggerate speech to make it more intelligible to get someone to hear or understand them. However, more of the isiZulu speakers versus the other language groups tended to pull down their masks when speaking if others could not hear them. This was statistically significant (*p* = 0.040; Pearson Chisq. test); however, the results must be viewed with caution given that the majority of the participants were isiZulu speakers. The majority, 76.0% (*n* = 73), felt that face masks and shields make it difficult to see facial expressions, and 60.4% (*n* = 58) reported difficulties to be heard clearly by others when they are wearing a mask, and most believed that it is difficult for one to be heard clearly by others when you are wearing a mask, this being statistically significant (*p* = 0.024; Fisher’s exact test).

### Objective 2

To determine health science students’ perceptions of the communicative impact of face coverings during the COVID-19 pandemic as listeners.

**FIGURE 2:**
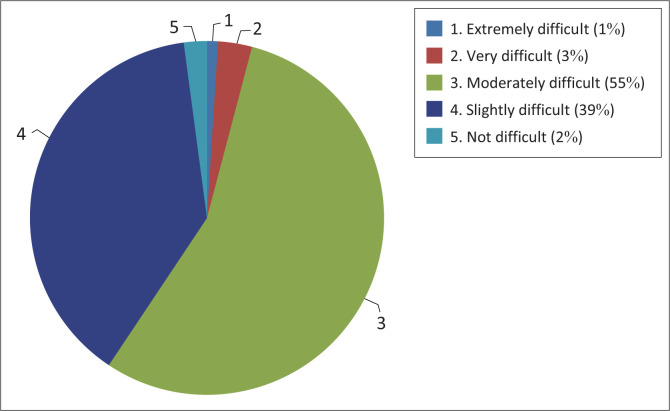
Level of difficulty in hearing a person speaking through a mask or shield (*n* = 96).

In this section, participants were asked to provide information on their level of difficulty in listening to people wearing face masks and shields. Overall, the majority, 55% (*n* = 53), of the participants reported that it was moderately difficult to hear a person who is speaking through a mask and shield, followed by 39% (*n* = 37) who said that it was slightly difficult to listen to a person who is speaking through a mask or shield (Figure 2).

Most 53.1% (*n* = 51) reported that they *always* use more listening effort when someone is using mask or shield, and 46.9% (*n* = 45) said they *sometimes* use effort when listening to someone wearing a mask or shield.

**FIGURE 3:**
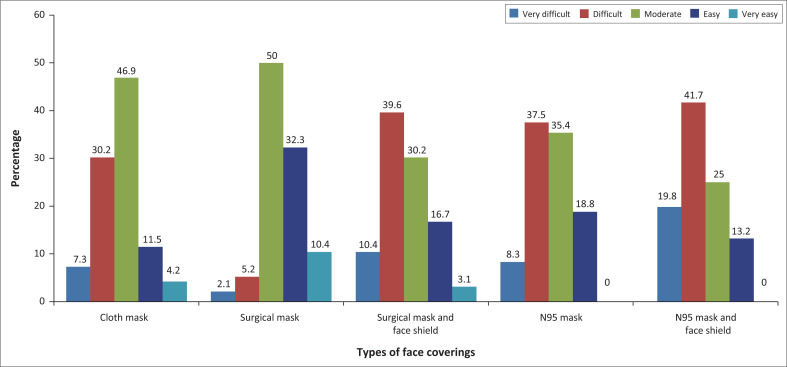
Difficulties in listening to a person wearing different types of face coverings (*n* = 96).

Most of them, 47% (*n* = 45), experienced moderate difficulty when someone was wearing a cloth mask, and 50% (*n* = 48) surgical mask. About 40% (*n* = 38) had difficulty when someone was wearing a surgical mask and shield, and 37.5% (*n* = 36) an N95 mask; 19.8% (*n* = 19) found it very difficult when both an N95 and face shield were used (see Figure 3). Most with self-report of poor hearing were more likely to say that it was difficult to hear when someone wore both a surgical mask and shield, with this being statistically significant (*p* = 0.009) (Fisher’s exact test).

A third, 33.3% (*n* = 32), of the participants reported that it was very difficult for them to see the other person’s mood (sad or unhappy) when wearing a mask; however, 27.1% (*n* = 26) reported that it was moderately difficult for them. A third, 33.3% (*n* = 32), also said it was moderately difficult to see if another person wearing a mask seemed happy or joyful, followed by 24% (*n* = 23) who reported that it was slightly difficult. Interestingly 11.5% (*n* = 11) of the participants said that it was not difficult, and equally, 11.5% (*n* = 11) reported that it was extremely difficult for them to see if a person who wore a mask seemed happy or joyful.

Regarding how they feel when they do not understand what the other person wearing a mask is saying, most 52.1% (*n* = 50) reported feeling anxious. In comparison, 45.8% (*n* = 44) reported that they feel frustrated, followed by 36.5% (*n* = 35) feeling stressed and overwhelmed, 24.2% (*n* = 23) felt embarrassed, and 23% (*n* = 21) who felt upset that they could not hear what the other person is saying. The majority said they felt anxious irrespective of language when communicating with someone wearing a mask. This was statistically significant (*p* = 0.037) (Pearson Chisq. test).

**FIGURE 4:**
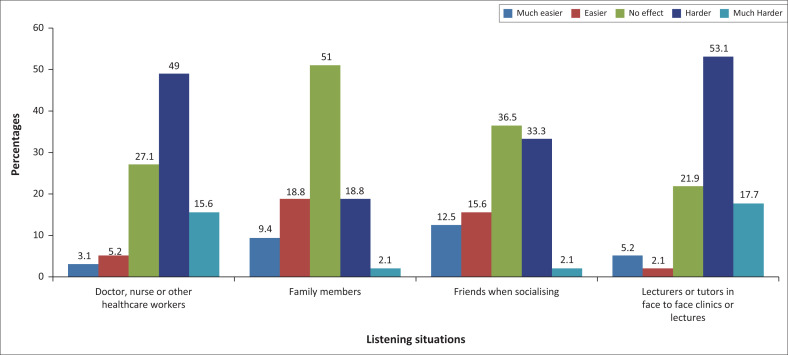
Impact of masks on the ability to hear and understand by the listening situation (*n* = 96).

Concerning the impact of face coverings by the listening situation on the ability to hear and understand, the majority, 51% (*n* = 49), reported no effect on their family members, followed by 36.5% (*n* = 34) who reported that there is no effect on their friends when socialising. Between 49% (*n* = 47) to 53% (*n* = 51) had difficulty with health workers and in lectures or clinic situations respectively (Figure 4). Most who were positive about wearing masks said it was harder to understand the doctor, nurse or other healthcare workers when they wore face coverings than when listening to their friends, which had little effect; this being statistically significant (*p* = 0.025; Fisher’s exact test).

**FIGURE 5:**
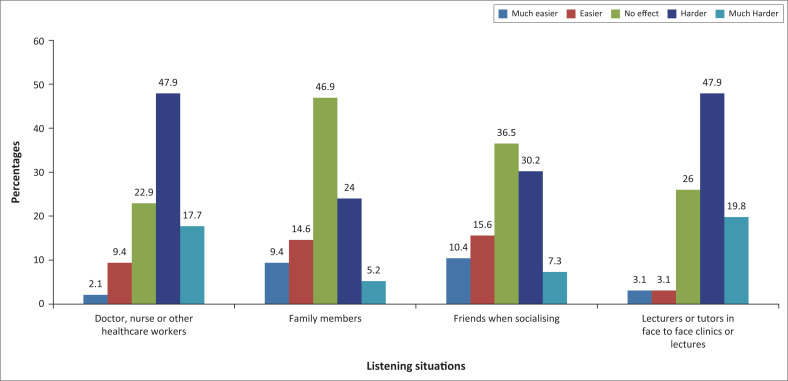
Difficulties in feeling connected and engaged with face masks being worn (*n* = 96).

The exact number of participants, 47.9% (*n* = 46), reported that it was harder for them to feel engaged and connected to the lecturers and tutors when they wore face coverings in face-to-face clinics or lectures and with doctors, nurses, and other healthcare workers. More of the participants, 46.9% (*n* = 45), said they had no effect with family members in terms of feeling connected or engaged, and 36.5% (*n* = 35) with friends when socialising (Figure 5). The third- and fourth-year students thought that it was more demanding than first- and second-year students to feel engaged in the conversation and feel connected to the speaker, especially healthcare professionals and lecturers versus friends and family. This was statistically significant (*p* = 0.033; Fishers exact test).

### Objective 3

To determine health science students’ perceptions of hearing and communicative impact of face coverings during the COVID-19 pandemic *in a clinical setting*.

Participants were provided two open-ended questions and were asked as future healthcare practitioners in their respective fields of study what they think would be the issues they will encounter in practice and strategies to mitigate the challenges on the communicative impacts of wearing masks. A total of 89/96 (*n* = 93%) commented on these. Fifty-six percent (*n* = 50/89), reported on potential communication challenges that could arise, including strained communication between patients and practitioners and personal discomfort. Emotional difficulties were reported by 20% (*n* = 18). Challenges related to COVID-19 pandemic, as alluded to by 24% (*n* = 21), related to issues around lack of social distancing and patient education, overcrowding, and so forth (see Table 3). Participants further provided various strategies to mitigate some of the challenges ranging from COVID-19-related strategies to control the spread of the virus to communication and environmental strategies. About 47% (*n* = 42/89) of participants emphasised the importance of adhering to COVID-19 protocols, with most indicating the use of face shields and vaccinations. About 37% (*n* = 33/89) further suggested that practitioners mainly speak louder and slower and use clear and transparent masks. Environmental strategies were proposed by 13% (*n* = 12) of participants (see Table 3).

**TABLE 3:** Challenges and strategies to mitigate these.

Envisaged communication challenges in practice (*n* = 89)	Strategies to mitigate challenges (*n* = 89)
**Communication difficulties:** 56% (*n* = 50/89) of participants responded. The main concerns centred around the following: Communication breakdowns between health workers and patients’ miscommunication of information Patients cannot connect and trust practitioners with the ‘barrier’ of a mask or a shield Difficulty communicating with patients who may rely on lip reading or speech reading Patients struggle to hear in background noise; they perceive speech as muffled and rely heavily on speech reading. Some patients can barely gather the strength to speak as it is if they have certain conditions- worse with masks Face masks make it even more difficult to communicate with patients High noise levels will be a problem Could issue wrong therapy or medication if can’t communicate and hear well Difficulty with kids and being able to see how they produce words	**Communication strategies:** 37% (*n* = 33/89) of participants responded. They suggested the following: Transparent masks that would make lip reading easier Surgical masks were better for breathing and communicating Practitioners should speak slowly, briefly, and articulate well Practitioners should be loud and clear. Face people when talking to them Speech and audiology practitioners should take a lead role in education and awareness creation. If persons with hearing loss or deafness are present, an interpreter should assist with communication. Use of videos, written word, and pictures to explain information Speak louder rather than getting people to come closer Have more online intervention instead of face-to-face interaction. Patients should be provided with speakers and mics. Counsel the patient we get the message across. Use nonverbal communication like hand gestures to demonstrate one’s points.
**COVID-19-related challenges:** 24% (*n* = 21/89) responded. The main concerns centred around the following: Patients not adhering to COVID-19 protocols Cross infection- people do not use masks and sanitise Most complained about lack of social distancing, which could be an issue for healthcare practitioners Some participants also alluded to areas of overcrowding Problems with patients adhering to COVID-19 prevention measures Patients must be encouraged to vaccinate	**COVID-19 strategies:** 47% (*n* = 42/89) of participants responded suggesting: Wear more protective gear and sanitise; Encourage vaccination; use clear shields and face masks; use N95 versus cloth or surgical masks, and use table shields. Clearly marked social distancing. Everyone needs to be screened, so their status is known; Reduce the number of people being attended to at the same time; more education to patients about COVID-19; Practitioners must wear surgical masks; Work rotations to minimise staff being in the same area all the time
**Emotional and personal challenges:** 20% (*n* = 18/89) responded The main concerns centred around the following: The use of masks affects communication and can lead to frustration. It affects the integrity of the intervention, challenges to building rapport Children also get intimidated by masks and other face coverings. Quite dehumanising wearing PPEs. We use face shields, but it is still scary and unsafe in COVID-19. Anxiety about contracting COVID-19 It also increases our workload as we have to repeat Lead to burnout and is very frustrating Physiotherapy is a hands-on profession- impractical to practice Increase workload and takes more time to repeat yourself Experience difficulty hearing our patients Hard for me to communicate with patients since some rely on facial expressions and lip reading to hear and understand speech As a spectacle wearer in the field of optometry, a mask or shield makes it harder for me to function as breath that escapes the mask from above clouds my glasses and my line of practices demands my vision to be clear as possible	**Environmental strategies:** 13% (*n* = 12/89) of participants responded as follows: Ensure good lighting when communicating. Less noise in the background Reduce overcrowding Patients should be booked on an appointment basis

PPE, personal protection equipment.

## Discussion

Participants in this current study adjusted the way they speak in relation to manner, content and length of utterances when wearing face coverings. This correlates with a study by Mheildy et al. (2020), where participants compensated by increasing the volume of speech and speaking more slowly to reduce the effect of muffled speech behind a facemask. The length of utterances is also affected by face masks, and positive outcomes are possible with communication when the length of utterances is changed (Bonnell, 2020). Additionally, a study by Asadi et al. (2020) found that people will adjust their speech or project their voice when using face coverings as these attenuate the sound. Exaggerated speech is more intelligible for someone to hear, and this speech cue seemed to enhance memory traces for sentences produced, enabling listeners to retain more information (Smiljanic, Keerstock, Meemann, & Ransom, 2021). A study by Goldin et al. (2020) also found that speech rate was lower for N95 and surgical masks, as speakers compensate to improve intelligibility. A study by Saunders et al. (2020) revealed that people misheard information during a conversation. Words are often misheard as masks reduce high pitch sounds where consonants that start or end with a high pitch sound are often misunderstood and therefore change the word’s meaning (Rahne, Fröhlich, Plontke, & Wagner, 2021). The use of face masks resulted in an increased vocal effort of the speaker, affecting the voice-breathing coordination, thereby limiting the overall communication and altering the perceptual features of the voice (Karagkouni, 2021).

Regarding interpersonal communication, most participants in the current study sometimes used bigger hand gestures and movements when speaking. A study by Mheidly et al. (2020) reports that people express their ideas using hand gestures to facilitate the communication process. Nonverbal communication can vastly influence the social environment, such as facial expressions, body movements and eye messages, which can support or substitute verbal communication (Mheidly et al., 2020). Participants stated that they focus more on eye contact when speaking. This correlates with a study conducted by Mheidly et al. (2020), which states that when wearing face masks, people focus more on the eyes to understand the other person’s facial expressions. However, one has to be cognisant that in certain cultures like the Zulu culture, eye contact is avoided as a sign of respect (Niba, 2011). However, according to Niba, (2011), in his study, he explains that the trend is slowly changing in cross-cultural South Africa as the benefits and advantages of eye contact were observed by participants and a growing acceptance that if healthcare workers maintained eye contact during the consultation process, it was polite as it assured them that the healthcare workers were paying attention to what they were saying. Eye contact can be used to show empathy and concern for others, manage feelings, express interest, or help with communication (Mheidly et al., 2020).

Most participants felt that face masks and shields make it difficult to see facial expressions. This correlates with a study by Chodosh et al. (2020), which states that with the use of face masks, one cannot access the facial expressions and lip movements vital to daily communication as face coverings prevent lip reading and muffle sound. Facial expressions play a critical role in communication, relaying important information that people perceive to help them predict events and situations and develop appropriate responses (Mheidly et al., 2020). The loss of visual and auditory information may be compensated by increased use of other information, such as a gentle nudge and visual clues that increase body language (Campagne 2021). Murphy et al. (2021) bring attention to dramatic arts, used in ancient Greece where actors used masks in theatres. The skills developed in the dramatic arts can be used in healthcare practitioner training, such as increasing the volume of voice, using hand gestures, using fewer words, and emphasising keywords to show care and empathy. Additionally, most participants stated that they pull their face mask down when they feel that the other person cannot hear or understand them. Similar findings were reported in a study by Ogoina (2020) were participants reported that they struggled with communication, engaging in a proper conversation and breathing when wearing a mask. They also reported that their words sounded muffled so they pull their mask down when talking. While most of the participants in the current study did not feel more self-conscious when speaking using face masks, about a third of participants felt more self-conscious when speaking using face masks. Communication stress emanates when interpersonal communication is negatively affected, as with face coverings (Campagne 2021), and can make you feel more distracted and self-conscious, further weakening your connection with others (Pryor, 2020).

Essentially, most of the participants in the current study reported some difficulties in hearing other people when they are wearing a mask which correlates with an investigation by Saunders et al. (2020). Participants had reported that face masks and coverings have an overall negative impact on hearing. In this current study, the results revealed that most participants felt moderately difficult in hearing someone speaking through a mask, while 40.6% reported that it was slightly difficult to hear someone speaking through a mask and shield. A possible reason for participants in this study only having moderate or slight difficulty could be that they are a group of young individuals who reported overall good self-reported hearing. A few studies that have examined the impact of face masks on communication have reported a mask-induced attenuation of the voice between 2 dB and 12 dB (Atcherson et al., 2017; Goldin et al., 2020; Mendel, Gardino, & Atcherson, 2008).

In this current study, most participants reported hearing difficulty when speaking with someone using an N95 mask and face shield and a surgical mask and face shield. In agreement with the statement above, a study by Rahne et al. (2021) revealed a greater listening effort in noise if the face masks are placed between the speaker and the listener. The face masks increased the absolute threshold of the hearing effort function, which reflects increased listening effort. The different types of masks worn may uniquely affect acoustic and speech perception, as masks differ in how they are composed and designed to sit on the person’s face. In a study by Toscano and Toscano (2021), the N95 respirator revealed the poorest speech recognition thresholds and speech intelligibility. Corey et al. (2020) and Magee et al. (2020) found that acoustic signals above 4 kHz were attenuated the most, regardless of the type of mask worn by the talker with disposable surgical face masks offering the best acoustic performance.

Most participants reported that it was difficult to understand the other person’s mood when wearing a mask, and some said it was extremely difficult to see a person’s mood while wearing a mask. A study by (Gabrielle, Jackson, & Visram 2021) reported that face masks impair people’s ability to classify emotional expressions accurately. Participants reported feeling anxious, frustrated, stressed and embarrassed when communicating. A study by Gabrielle et al. (2021) found that the communication issues associated with face coverings elicited a diverse array of negative emotions, including anxiety, isolation, feeling stupid and losing confidence. Some clinicians also experience adverse psychological symptoms associated with mask-wearing (Marler & Ditton, 2021; Xiong et al., 2020). The mouth transmits the emotional content and meaning of the message, and one can already perceive messages from the speaker’s mouth, such as happiness, sadness, anger and doubt. Facial recognition is thus an important social and psychological input for both children and adults (Campagne 2021).

Most participants reported that it was harder for them to feel engaged/connected to the lecturers, tutors, doctors, nurses, and other healthcare workers than their friends and family. A study by Saunders et al. (2020) found that the reported impacts of face coverings vary by listening situation. The implications of communicating in a healthcare setting are more significant than communication with families or friends. This could be interpreted as suggesting that the perceived impact of the face covering is associated with some combination of the importance of information being discussed, the familiarity of the person or people speaking, and the predictability of the content of the discussion, rather than solely the acoustic environment in which communication is taking place. It is not surprising then, those healthcare situations in which a relatively unfamiliar individual often shares essential information in an already stressful situation are particularly anxiety-provoking (Saunders et al., 2020).

Most participants commented on the communication breakdowns between health workers and patients because of strained communication. This leads to more communication errors, multiple repetitions and more listening effort as a result of using face coverings compounded by noise levels (Bandaru et al., 2020). With the increased workload, effective communication between practitioners and patients is essential to ensure that health care is delivered effectively (Bandaru et al., 2020). Effective communication is a central necessity in building therapeutic relationships, with functional professional relationships a prerequisite in providing high-quality care (Ha & Longnecker, 2010). A study conducted by Altschuler (2021) found that patients, healthcare providers and caregivers experienced serious communication challenges both within and outside the hospital. The use of a face mask compromises communication, especially in competing noise (Yi et al., 2021). Face masks obscure facial expressions and communication and may affect the doctor–patient relationship and overall treatment outcome (Padhy, Rina, & Sarkar 2020). When healthcare practitioners cannot show emotional rapport and expression, the goal of the clinical encounter will not be fulfilled. It will be hard to build relationships, promote rapid trust, encourage information sharing, and show compassion and concern (Padhy et al., 2020). Expressions of emotion and reciprocity of nonverbal communication serve as important foundations for clinical encounters (Houchens & Tipirneni, 2020).

Many of the suggestions provided by the participants to the challenges they envisage in practice are similar to those in a study by Mheidly et al. (2020) related to raising awareness about the impact of face coverings on communication, emphasising nonverbal communication, concentrating on speech rate and loudness, paying attention to noise levels in a different setting, and use of telecommunication and online strategies. It is anticipated that strategies such as greater use of nonverbal cues, using a raised voice and other coping mechanisms may be helpful for speakers and listeners in enhancing intelligibility in conditions that require face coverings to be worn and may ease communication challenges (Saunders et al., 2020). Lapel or clip-on microphones may be used in conjunction with transparent plastic window masks, which will allow visual cues without destroying the high-frequency sounds where the lapel microphones are placed above and below the mask. This combination has been recommended by Corey et al. (2020). Participants also suggested more online work, including for an example tele therapy, speaking louder and slower when communicating and writing down information to get the message across to patients. The use of videos and pictures to explain information and write down things is also supported by a study carried out by Simpe (2021). Practitioners can be responsible for developing and circulating well-informed, consistent communication guides for all patients and practitioners experiencing communicative challenges, exacerbated by mask wearing (Marler & Ditton, 2021; Martinelli et al., 2021).

## Limitations, implications and recommendations

Limitations of online surveys include not having stable internet and data connection; control over participants’ answers are limited and response rates may be low. This study was conducted at one university only the interpretation is limited to the study site. Therefore, the findings cannot be generalised.

Some clinical implications resulting from the study findings necessitate more education and training students in health science disciplines and possibly the wider university community on the use of face coverings and shields and communication impact. This includes more education and information on the effects that different face masks and shields have on hearing and communication and the impact on interpersonal communication, ways in which communication breakdowns can occur with face masks and shields, and how to mitigate these. Research implications could include how patients with hearing loss receive appropriate healthcare information and how they adhere to instructions given by the audiologist when both are wearing masks and face coverings. In South Africa, we have patients from various cultural and language backgrounds and dialects; the added challenge posed by face coverings needs to be explored further. Implications of how communication is affected when wearing face coverings on students receiving the lecture content when lecturers wear a mask need to be researched. Perhaps, a larger study needs to be conducted, including other health sciences disciplines from other universities, to obtain a more comprehensive view of face coverings, shields and communication impact, and how to mitigate discipline-specific challenges. Studies need to be conducted utilising interviews as opposed to questionnaires as this would allow more in-depth, accurate reflections on perceptions, feelings and attitudes as opposed to self-administered questionnaires.

## Conclusion

The COVID-19 pandemic has changed the face of healthcare delivery globally. Everyone is now expected to wear a mask or face covering, which is likely to be a feature of patient care for a long time to come. However, face coverings while being protective against the virus are also known to impact communication. Health science students are future health practitioners who need to understand the communicative impacts of face coverings. All participants in this current study changed their manner of speaking when wearing masks and focused more on eye contact when someone was wearing masks and they used more listening effort. They reported feeling anxious when communicating and found it challenging to read emotions. The level of difficulty differed depending on the listening environment. Most demonstrated good understanding of challenges they could face as future practitioners and provided some strategies to mitigate these. Therefore, it is envisaged that improved understanding, increased awareness and practical strategies on clear, effective communication will assist in guiding patient-practitioner communication and interaction during the pandemic and beyond.
